# Resveratrol and Amyloid-Beta: Mechanistic Insights

**DOI:** 10.3390/nu9101122

**Published:** 2017-10-14

**Authors:** Yongming Jia, Na Wang, Xuewei Liu

**Affiliations:** 1 Department of Neuropharmacology, Research Institute of Medicine and Pharmacy, Qiqihar Medical University, Qiqihar 161006, China; yongmingjiahlj@126.com; 2 Department of Pathophysiology, Qiqihar Medical University, Qiqihar 161006, China; christina85721@163.com

**Keywords:** resveratrol, amyloid-beta, alzheimer disease, transporter, blood-brain-barrier

## Abstract

The amyloid-beta (Aβ) hypothesis that dyshomeostasis between Aβ production and clearance is a very early, key molecular factor in the etiology of Alzheimer’s disease (AD) has been proposed and examined in the AD research field. Scientists have focused on seeking natural products or drugs to influence the dynamic equilibrium of Aβ, targeting production and clearance of Aβ. There is emerging evidence that resveratrol (Res), a naturally occurring polyphenol mainly found in grapes and red wine, acts on AD in numerous in vivo and in vitro models. Res decreases the amyloidogenic cleavage of the amyloid precursor protein (APP), enhances clearance of amyloid beta-peptides, and reduces Aβ aggregation. Moreover, Res also protects neuronal functions through its antioxidant properties. This review discusses the action of Res on Aβ production, clearance and aggregation and multiple potential mechanisms, providing evidence of the useful of Res for AD treatment.

## 1. Introduction

Alzheimer’s disease (AD) is considered the most frequent form of dementia, affecting more than 35.6 million people worldwide and becoming an emerging burden on society [1,2]. Although researchers have delved into all aspects of this complex, multifactorial syndrome in recent years, the exact molecular events underlying AD remain to be exhaustively elucidated. The amyloid-beta (Aβ) hypothesis which synthesizes histopathological and genetic information has become the dominant model of AD pathogenesis and is guiding the development of a novel strategy in the treatment of AD [3]. The pathological accumulation of Aβ in the brain leads to impairment of synaptic function and structure, progressive tau deposition, neuronal destruction and finally the clinical symptoms of AD [3]. Aβ homeostasis in the brain is governed by its production and clearance mechanisms [4]. Aβ was produced by sequential scission of amyloid precursor protein (APP) by the β-APP cleaving enzyme (BACE) and γ-secretase [5]. Numerous pathways have been reported to play roles in Aβ clearance and degradation, including autophagic pathway and receptor-mediated endocytosis [6]. Some proteins with a crucial role in Aβ clearance are Aβ-degrading enzymes (ADEs), which could degrade or cleave Aβ into smaller fragments, and lipoprotein receptor-related protein 1 (LRP1), which regulates metabolism of Aβ and brain homeostasis through multiple pathways and transporters such as P-glycoprotein (P-gp), localized in astrocytes and on the abluminal side of the cerebral endothelium, where they transport Aβ from brain to blood [7,8,9]. So some researchers focused on screening drugs which can influence the dynamic equilibrium of Aβ, targeting production and clearance of Aβ.

Resveratrol (3,5,4’-trihydroxy-trans-stilbene, Res), a naturally polyphenolic phytoalexin, abundantly found in grapes, berries, red wine and many other plant species, shows diverse biological activities such as antioxidant, anticarcinogenic and anti-inflammatory properties, and so on [10]. Recent evidence found that, in various in vitro and in vivo models of AD, Res plays a prominent role in the prevention and treatment of AD [11,12]. Res attenuated Aβ-induced cytotoxicity, apoptosis and intracellular reactive oxygen intermediate (ROI) accumulation in PC12 cells [13]. Similarly, Res inhibits Aβ-induced neuronal apoptosis in PC12 cells through regulation of silent information regulator 1 (SIRT1)-Rho-associated kinase 1 (ROCK1) signaling pathway [14]. In Res-treated APP/PS1 mice which are a model of cerebral Aβ deposition, there was a significant reduction in the number of activated microglia, providing anti-inflammatory effects of Res against Aβ-triggered microglial activation [15]. It is interesting that Aβ accumulation in the brain has been shown to induce cytotoxicity, apoptosis and activation of astrocytes in cell and animal models [16,17,18]. So we explore whether Res can affect Aβ accumulation and aggregation in AD model (Table 1 and Table 2). In this review, we discuss the effects of Res on Aβ production and clearance and clarify those multiple mechanisms in various models and explore Res as a new drug candidate in AD treatment.

## 2. The Source and Pharmacological Profile of Res

**Figure 1:**
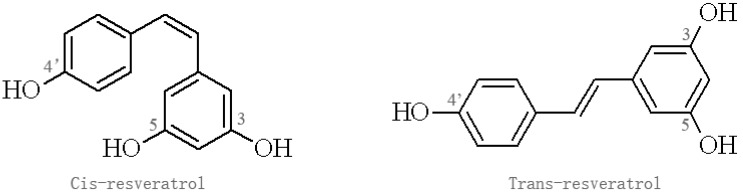
Isomers of resveratrol.

Res belongs to the stilbene family of phytoalexins produced by various plants in response to environmental stress, which have been detected in at least 72 plant species, especially grape vines, pine trees, jackfruit, blueberry, cranberry, and mulberry [19,20]. Res containing two aromatic rings in its structure exists in two isomers: trans-Res, which has been most widely studied, and *cis*-Res; the trans form is more stable and potent than the *cis* form, which may have different biological effects (Figure 1) [21]. Res has diverse biological activities and is not known to cause significant adverse effects in experimental animals and humans [22,23,24]. A clinical trial found that oral Res at a single dose of 5 g in 10 healthy volunteers resulted in no apparent adverse effects, and the peak level of Res in plasma was 539 ng/mL, while plasma concentration of two Res monoglucuronides and Res 3-sulfate were 1285 ng/mL, 1735 ng/mL, 4294 ng/mL, respectively [22]. Similarly, a double-blind, randomized, placebo-controlled study showed that Res is safe in healthy individuals, but produced a relatively low plasma concentration. Res exhibits low oral bioavailability due to its rapid metabolism and elimination, which is rapidly metabolized by conjugation to glucuronic acid and/or sulfate, forming Res glucuronides, sulfates, and/or sulfoglucuronides [25]. Thus, Res analogues and inhibitors affecting Res metabolism were developed to enhance its bioavailability.

## 3. Effect of Res on Aβ Production

### 3.1. Inhibitory Activity against BACE1

BACE1, mainly expressed in neurons of the brain, exhibited all the known characteristics of the β-secretase, which cleave extracellular of APP important in the pathogenesis of AD [36]. Tamagno et al. reported that Aβ induces production of oxidative stress markers 4-hydroxynonenal and upregulates BACE-1 expression in NT2 neuronal cells, further fostering amyloidogenic processing of APP, thereby increasing accumulation of Aβ [37]. Scientists hope to find some active compounds or develop new drugs to block BACE1 (BACE inhibitors), further decreasing Aβ accumulation, regarding BACE as a key target for AD treatment. Studies showed that Res and Res oligomers significantly inhibited BACE activity in a dose dependent manner, which was assessed by fluorescence resonance energy transfer (FRET) assay [30]. Similarly, Koukoulitsa et al. found that Res and its derivatives bearing one (*tert*-butyl, 1-ethylpopyl) or two bulky electron donating groups ortho to 4’-OH displayed different potencies against BACE1 using time-resolved fluorescence (TRF) assay, further suggesting that Res can inhibit BACE1 function [31,38]. In contrast, Marambaud et al. found that Res does not inhibit Aβ production through neither affecting β- nor γ-secretases [39]. The paradoxical results may be caused by different methods and cells, which need further studies. Some potent BACE1 inhibitors is now undergoing clinical trials such as E2609, AZD3293 and LY2886721, and some adverse effects also reported [40]. Finding active components such as Res in herbs targeting β-secretases could be a strategy in AD treatment.

### 3.2. Inhibitory Activity against γ-Secretase

γ-Secretases are a family of intramembrane cleaving aspartyl proteases, consisting of four subunits presenilin (PSEN), anterior pharynx defective-1, nicastrin, and presenilin enhancer-2 [41]. When the expression and activity of those four subunits were changed, the catalytic function of γ-secretases could also be changed [42]. Recent evidence showed that rats fed with Res, a SIRT1 inducer, exhibit a significant increase in PSEN1 expression, which is one of SIRT1-specific DNA targets [26]. Unfortunately, the results did not show whether the activity of γ-secretase was changed and Aβ production could be suppressed by SIRT1 [26]. Choi et al. found that Res (5–20 μM) as well as its analogues inhibit γ-secretase activity and increase α-secretase activity in Neuro2a neuroblastoma cells, which may be associated with a decrease in Aβ levels, without causing cell death [32]. However, another study showed that Res has no effect on γ-secretases, without affecting Aβ production and APP metabolism [39].

### 3.3. Autophagy Induction

Emerging evidence indicated that autophagy pathways could affect developmental and neurodegenerative processes, including AD. AD etiology is also associated with damaged mitochondria in the neurons. Misfolded proteins translocated and accumulated into the mitochondrial membrane, leading to the disruption of oxidative phosphorylation and then autophagy activation [43]. Accordingly, neuronal autophagy is known to play key roles in synaptic plasticity, anti-inflammatory function in glial cells, oligodendrocyte development, and myelination process [44]. Studies found that a decline in autophagy efficiency during aging led to accumulation of Aβ and cytochrome c release in the mitochondrial membrane, resulting cell death and neurodegeneration [45,46]. Moreover, accumulation of pathological autophagic vacuoles can be observed in PS1/APP mouse AD model [47]. Imbalance between autophagic flux and degradation result in decreased proteolysis of Aβ [48]. In AD, autophagy impairment stimulates PSEN1 expression and then increases γ-secretase activity, leading to augment of Aβ synthesis [49]. Res, an autophagy inducer, decreased PSEN1 expression, consistent with a suppression of Aβ production [33]. Moreover, Res can activate tyrosyl transferRNA (tRNA) synthetase (TyrRS)-auto-poly-ADP-ribosylation of poly (ADP-ribose) polymerase 1 (PARP1)-SIRT1 signaling pathway, inducing autophagy in PC12 cells, thereby attenuating neurotoxicity caused by Aβ [50]. Res have the therapeutic potential of AD through activating autophagy.

## 4. Effect of Res on Aβ Clearance

Metabolic pathway of Aβ in AD pathogenesis, especially in late-onset sporadic AD (LOAD) has been raised, and the main mechanisms of Aβ clearance have been considered as new therapeutic targets [51,52]. Res could modulate Aβ clearance in various ways such as activation of ADEs, transport across the blood-brain barrier (BBB) into the circulation, uptake by microglial phagocytosis and inhibition of Aβ aggregation [53,54].

### 4.1. Activation of ADEs

Disturbances in the activity of ADEs, including neprilysin (NEP), insulin-degrading enzyme (IDE), angiotensin-converting enzyme (ACE), endothelin-converting enzyme (ECE) and plasmin, could induce Aβ accumulation, resulting in AD pathology [55,56]. Activity of ADEs could be affected by Res in different AD models. In vitro studies, NEP and ACE activities were induced effectively in SK-N-SH cells with low concentrations of Res for 4 days [34]. The authors found that Res enhances the differentiation state of proliferating cells, which correlated with up-regulation of the cellular enzymatic activity to enhance NEP and ACE activity [34]. Similarly, Marambaud found that NEP activity was significantly increased after Res treatment on intact HEK293 cells. However, proteasomal degradation pathway might involve in Res-mediated decrease of Aβ but not the increased NEP activity induced by Res [16]. Moreover, study found that in human endothelial cells, Res decreased ECE-1 mRNA expression and reduced ECE-1 activity, further affecting endothelin synthesis and conversion; however, incubation concentration of Res (30 μM) cannot achieve in rat and/or human plasma [57]. Although ECE-1 activity can be inhibited by Res, it cannot induce Aβ accumulation in brain, leading to AD. In vivo study, Res could dramatically increase both the estradiol level and NEP level in LPS-treated rats and control rats using NEP ELISA kit [27]. Those results indicated that Res can involve in activation of ADEs, but whether Res promotes clearance of Aβ through ADEs needs further studies.

### 4.2. Plasminogen System

The plasminogen system is a complex enzymatic cascade [58]. Plasmin (PL), degrading many plasma proteins, is an important serine protease released from the inactive zymogen plasminogen (PLG) by two physiological activators tissue-type plasminogen activator (t-PA) and urokinase-type plasminogen activator (u-PA) [59]. Function of t-PA and u-PA were controlled by PA inhibitor type 1 (PAI-1) and type 2 (PAI-2) [60]. PL is involved in many pathophysiological processes through its ability to cleave fibrin, fibronectin, thrombospondin, laminin, and von Willebrand factor. The plasminogen system has been implicated in neuronal plasticity and long-term potentiation (LTP) in the brain [61]. Studies showed that tPA-plasmin system contributes to the clearance of Aβ in mouse brain and the prevention of Aβ-induced neurotoxicity, suggesting that the plasminogen system is associated with late-onset AD [62]. Similarly, polymorphisms in the u-PA gene have been associated with AD susceptibility [63]. Res increased plasminogen activators t-PA and u-PA expression in cultured human umbilical vein endothelial cells (HUVECs), leading to plasminogen endoproteolysis and plasmin activation.

### 4.3. Neurovascular Pathways

The neurovascular unit (NVU) consists of different cell types, including brain endothelial cells, glial cells and neurons, which controls BBB permeability, neurovascular coupling and clearance of toxins such as Aβ from brain [64]. BBB permeability controls entry from blood and promotes clearance of macromolecules from the brain [64]. Moreover, transporters and receptors in the brain endothelium regulate delivery of drugs and endogenous substances in the brain. Studies have shown that Aβ is a substrate of LRP1, the receptor for advanced glycation end products (RAGE) and several ABC transporters such as P-gp and Breast cancer resistance protein (BCRP) [65,66,67,68]. Under physiological conditions, vascular damage and changes in the expression of several BBB transporters and receptors could affect Aβ transport from blood-to-brain and/or brain-to-blood. Res, a novel transporter modulator, defends BBB integrity and changes transporter and receptors expressions in BBB, thereby regulating Aβ homeostasis [28,65]. We will focus on effect of Res on: (1) integrity of BBB, which regulates delivery of energy metabolites and essential nutrients; (2) ABC transporters in brain that mediate Aβ efflux into circulation from brain; (3) LRP1 mediating Aβ efflux from brain; and (4) RAGE, which mediates Aβ reentry into the brain from circulation and the neurovascular inflammatory response.

#### 4.3.1. BBB Integrity

Several studies have suggested that injured BBB integrity and cerebrovascular dysfunction lead to faulty Aβ clearance from the brain [69]. Res could protect BBB integrity in different animal models [70]. In ovariectomized + D-galactose induced rat model of AD, Res decreases the insoluble Aβ_42_ level and protects the BBB integrity through regulating the expressions of RAGE, matrix metalloprotein-9 (MMP-9) and Claudin-5 [29]. In clinic, Res appears to restore BBB integrity in AD patients, reducing the ability of harmful immune molecules secreted by immune cells to infiltrate from the body [71]. Similarly, Res has exhibited restoration of BBB integrity via reduction of MMP-9 and induce adaptive immune responses which may promote brain resilience to Aβ deposition in AD patients [72]. Those studies suggested that Res could protect BBB integrity, changing Aβ homeostasis.

#### 4.3.2. P-gp

In vitro and in vivo studies showed that eliminated P-gp, first ABC transporter detected in endothelial cells of the human BBB lead to Aβ accumulation [9,73]. Furthermore, P-gp activity at the BBB is reduced in AD individuals [74], suggesting that impaired P-gp activity may mediate cerebral Aβ accumulation. Our previous studies indicated that Res enhanced bestatin absorption by downregulating P-gp expression in Caco-2 cells [10]. Moreover, Res and its major metabolites could penetrate BBB and be measured in Cerebrospinal fluid (CSF) in AD patients [75], at least indicating that Res has the opportunity to have CNS effects. Unfortunately, no effects of Res treatment on Aβ_42_ level in plasma and CSF has been detected [75], and no research has yet investigated whether P-gp expression at the BBB is changed by Res. A larger study is required to determine whether Res can affect Aβ clearance through regulating P-gp.

#### 4.3.3. LRP1

LRP1, one receptor in the LDL receptor family, is not only a multifunctional scavenger and cargo transporter but also has signal transduction activity [76]. As a cargo transporter, some studies have reported that LRP1 transported several ligands from brain to blood including Aβ [77,78]. Moreover, several genetic studies have indicated that LRP1 expression at the BBB is reduced in AD and have been considered as a therapeutic target in AD [79,80]. After treatment with Res in AD transgenic female mice, the brain LRP1 protein expression was increased, while its mRNA was not changed [28]. The author further found that Res could upregulate and stabilize transthyretin (TTR) which binds Aβ peptide avoiding its aggregation and toxicity, resulting increased LRP1 [28]. In vitro studies, LRP1 mediated the cytotoxicity of the fibrillar Aβ oligomers following their binding to prion protein (PrP^C^), and Res could reduce PrP^C^ and LRP1-mediated binding in SH-SY5Y cells, remodeling the fibrillar Aβ oligomer conformation [81].

#### 4.3.4. RAGE

RAGE, a multiligand receptor of the immunoglobulin superfamily of cell surface molecules, binds distinct classes of ligands such as AGE proteins and Aβ [82]. RAGE expressed at the luminal side of the BBB transport Aβ from the blood into the brain. In addition, intraneuronal transport of Aβ via neuronal RAGE leads to mitochondrial dysfunction [83]. RAGE expression is increased in the AD capillary under pathological conditions. Res has been shown to reduce RAGE expression in vascular cells [84]. Scientists are now comfortable referring to AD as type 3 diabetes, which results from insulin resistance in the brain. Similarly, Res can also downregulate RAGE expression in the kidney and liver of rats with type 2 diabetes [85,86]. However, whether RAGE expression triggered by Res could affect Aβ uptake, leading to Aβ accumulation in brain needs further research. Given the important role of RAGE in Aβ accumulation in AD, finding drugs which can block RAGE may contribute to control of Aβ-mediated brain disorder.

## 5. Aβ Plaque Disruption

Aggregation of Aβ leads to activation of microglia and astrocytes, and loss of cholinergic neurons, which is a constant feature of AD [87]. Res not only plays an important role in affecting Aβ homeostasis, but can also inhibit Aβ aggregation from lower molecular weight oligomers into higher molecular weight oligomers and disrupt preformed Aβ aggregation [54,88]. One study found that Res could bind directly to Aβ in different states including monomer and fibril Aβ [35]. Additionally, from indirect inhibitory effect, Res could enhance the binding of Aβ oligomers and TTR which can stabilize Aβ oligomers structure, preventing plaque aggregation, which provides new perspective into the protective properties of Res against AD [28,89].

## 6. Res Analogs in AD Treatment

Res analogs which show better bioavailability, efficacy and stability compared to Res are being tested for their activity in relation to many degenerative conditions. Some studies showed that Res derivatives exhibit neuroprotective effects in vivo and in vitro models [90]. Compounds 5d, a3, 5-dimethoxyl derivatives of Res, can cross BBB in vitro and are the potent inhibitor of Aβ_42_ aggregation, disintegration of highly structured with low neurotoxicity [91]. Similarly, Piceatannol, a metabolite of Res found in red wine, protects Aβ-induced neural cell death through affecting the accumulation of reactive oxygen species (ROS) induced by Res in PC12 cells [90]. Pterostilbene is a stilbenoid chemically related to Res and is a potent modulator of cognition and cellular stress, associated with regulating peroxisome proliferator-activated receptor alpha (PPARα) protein expression [92]. Various Res analogs and derivatives developed with improved bioavailability possess neuroprotective activities and could be promising drug candidates in the treatment of AD.

## 7. Conclusions and Challenges

**Figure 2:**
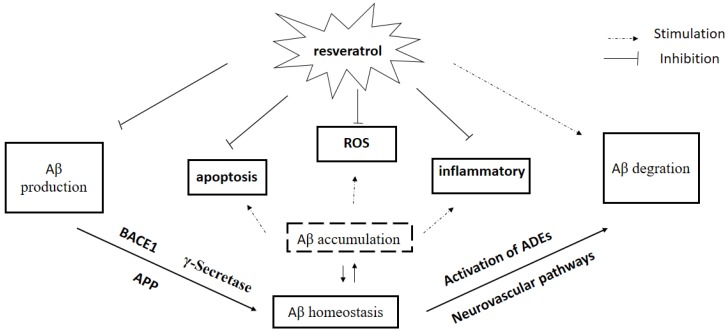
Effect of resveratrol on Aβ homeostasis. Aβ: amyloid-beta; APP: amyloid precursor protein; ROS: reactive oxygen species; BACE: β-APP cleaving enzyme. ADEs: Aβ-degrading enzymes.

Recent observations provide strong evidence for the link between Aβ accumulation in the brain and AD, and the role of Aβ clearance pathway in AD. There is an urgent need to search for and develop disease-modifying natural products and drugs to treat the major neurodegenerative disorders [93]. In this chapter, we have briefly reviewed the literature on effects of Res, a new rising star in AD treatment, on Aβ production and Aβ clearance, BBB dysfunction, and Aβ plaque disruption, supporting an essential role of Res on Aβ homeostasis in AD pathogenesis (Figure 2). In addition to directly regulating Aβ homeostasis in brain, several in vitro studies showed that Res is believed to afford strong antioxidative and anti-inflammatory properties induced by Aβ, indirectly. Res attenuated Aβ-induced oxidative stress in vivo and in vitro [94,95], suggesting that Res may be of benefit in AD treatment. Clinical trials indicated that Res seems to be well tolerated with less toxicity and can penetrate BBB easily. However, low oral bioavailability of Res limited its clinical efficacy because of rapid excretion and extensive metabolism [96]. Numerous clinical trials tried to investigate the effects of Res on neurodegenerative diseases including AD, although there are many difficulties such as bioavailability and side effects [97]. Addressing these questions will lead to a better understanding of mechanisms of Res on AD treatment, which will contribute to searching for, developing and designing new drugs for AD.

## Figures and Tables

**Table 1:** Neuroprotective effects of resveratrol in Alzheimer’s disease (AD) models in vivo.

Experimental Animal	Model/Method	Action of Resveratrol	Dosage	Duration of Treatment	Reference
APP/PS1 mice	AD	Inhibits Aβ-mediated microglial activation	AIN-93G diet supplemented with 0.35% resveratrol	15 weeks	[15]
Sprague-Dawley rats	-	increases PSEN1 expression in the rat brain	dietary resveratrol	28 days	[26]
Mice	Injected with lipopolysaccharide	increases both the estradiol level and NEP level	injected with resveratrol 4 mg/kg	7 days	[27]
APP/transthyretin (TTR) mice	AD	Increased LRP1 expression upregulated and stabilizes TTR	174 mg/kg/day	2 months	[28]
Wistar female rats	AD	decreases level of insoluble Aβ in the hippocampus	80 mg/kg	12 weeks	[29]
		reduces the expression of RAGE in the hippocampus			
		inhabits the expression of MMP-9			

Aβ: amyloid-beta; NEP: neprilysin; APP: amyloid precursor protein; LRP1: lipoprotein receptor-related protein 1; RAGE: the receptor for advanced glycation end products; MMP-9: matrix metalloprotein-9.

**Table 2:** Mechanism of resveratrol on amyloid-beta (Aβ) production and clearance in vitro.

Experimental Model/Method	Exposure	Mechanism of Resveratrol	Dosage of Resveratrol	Duration of Resveratrol Treatment	Reference
PC12 cells	Aβ_25–35_ induced	attenuated Aβ-induced cytotoxicity, apoptotic features, and intracellular ROI accumulation.	25 μM	24 h	[13]
PC12 cells	Aβ_25–35_ induced	inhibited the cell apoptosis	12.5–100 μM	24–48 h	[14]
		prevented the LDH leakage			
		maintained the intracellular Ca^2+^ homeostasis			
Purified baculovirus-expressed BACE-1	-	inhibition of BACE-1	11.9 μM (IC_50_)	-	[30]
TRF assay	-	inhibition of BACE-1	28 μM (IC_50_)	30 min	[31]
Neuro2a cells HEK293	transfected with a plasmid containing APPsw	reduced γ-secretase activity	2.5–20 μM	24 h	[32]
		induced MMP-9 activation			
autophagy-related 5 knockdown HEK293	-	induced conversion of LC3-I to LC3-II	60 μM	24 h	[33]
		suppression of Presenilin-1 induction			
		suppressed Aβ production			
SK-N-SH cells	-	induction of NEP and ACE activity	10 μM	4 days	[34]
Hippocampal samples from AD patients	AD	binds to both fibril and monomer Aβ	1.56–100 μM	-	[35]

ROI: reactive oxygen intermediate; BACE: β-APP cleaving enzyme.
